# Continuous cardiac thermometry via simultaneous catheter tracking and undersampled radial golden angle acquisition for radiofrequency ablation monitoring

**DOI:** 10.1038/s41598-022-06927-9

**Published:** 2022-03-07

**Authors:** Maxime Yon, Marylène Delcey, Pierre Bour, William Grissom, Bruno Quesson, Valéry Ozenne

**Affiliations:** 1 IHU Liryc, Electrophysiology and Heart Modeling Institute, Hopital Xavier Arnozan, Foundation Bordeaux Université, Avenue du Haut Lévêque, 33604 Pessac Cedex, France; 2 Centre de Recherche Cardio-Thoracique de Bordeaux Inserm, U1045, Université de Bordeaux, 33000 Bordeaux, France; 3 INSERM, Centre de recherche Cardio-Thoracique de Bordeaux, U1045, F-33000 Bordeaux, France; 4 Department of Biomedical Engineering, Vanderbilt University, 5824 Stevenson Center, Nashville, TN 37235 USA

**Keywords:** Cardiac device therapy, Imaging techniques

## Abstract

The complexity of the MRI protocol is one of the factors limiting the clinical adoption of MR temperature mapping for real-time monitoring of cardiac ablation procedures and a push-button solution would ease its use. Continuous gradient echo golden angle radial acquisition combined with intra-scan motion correction and undersampled temperature determination could be a robust and more user-friendly alternative than the ultrafast GRE-EPI sequence which suffers from sensitivity to magnetic field susceptibility artifacts and requires ECG-gating. The goal of this proof-of-concept work is to establish the temperature uncertainty as well as the spatial and temporal resolutions achievable in an Agar-gel phantom and in vivo using this method. GRE radial golden angle acquisitions were used to monitor RF ablations in a phantom and in vivo in two sheep hearts with different slice orientations. In each case, 2D rigid motion correction based on catheter micro-coil signal, tracking its motion, was performed and its impact on the temperature imaging was assessed. The temperature uncertainty was determined for three spatial resolutions (1 × 1 × 3 mm^3^, 2 × 2 × 3 mm^3^, and 3 × 3 × 3 mm^3^) and three temporal resolutions (0.48, 0.72, and 0.97 s) with undersampling acceleration factors ranging from 2 to 17. The combination of radial golden angle GRE acquisition, simultaneous catheter tracking, intra-scan 2D motion correction, and undersampled thermometry enabled temperature monitoring in the myocardium in vivo during RF ablations with high temporal (< 1 s) and high spatial resolution. The temperature uncertainty ranged from 0.2 ± 0.1 to 1.8 ± 0.2 °C for the various temporal and spatial resolutions and, on average, remained superior to the uncertainty of an EPI acquisition while still allowing clinical monitoring of the RF ablation process. The proposed method is a robust and promising alternative to EPI acquisition to monitor in vivo RF cardiac ablations. Further studies remain required to improve the temperature uncertainty and establish its clinical applicability.

## Introduction

Catheter ablation under X-ray fluoroscopy is one of the reference methods for the treatment of arrhythmia^1^. Over the past 20 years, tremendous progress^2^ have been made in the integration of imaging techniques in these procedures^3,4^. The utility of image guidance for atrial fibrillation^5^ or ventricular tachycardia^6^ ablations has been demonstrated at all stages of the procedures. Nevertheless, clinical outcomes remain conditioned by inherent limitations of the X-ray fluoroscopy modality which cannot depict in real-time the energy delivery to the tissue or the extent of the lesion. Although these markers are governed by the transfer of radiofrequency (RF) energy to the tissue, physiological factors such as the intermittent contact between the catheter and the tissue, the myocardial electrical impedance, the tissue perfusion, the tissue orientation, and the blood flow in the cavity lead to large variability and prevent accurate prediction of the final lesion size clinically^7^.

To overcome this limitation, real-time MRI guidance for cardiac ablative treatment of arrhythmia has, over the last decade, moved from the initial proof of concept^8–10^ to first clinical studies^11^. MRI has the potential to add value for substrate identification^12–14^ and lesion evaluation^15^, which can avoid to redo procure due to incomplete treatment. MRI also offers several contrasts such as T_1_^16^, T_2_^17^ LGE, TI-long^18,19^ to assess either the myocardium viability or acute/chronic lesion sizes. Moreover, it offers the ability to monitor in vivo and non-invasively the tissue temperature and thus lesion formation in real-time using Magnetic Resonance Temperature Imaging (MRTI)^20–22^. The proton resonance frequency (PRF) shift^23–25^ is the most well-established MRTI method due to its compatibility with fast and simple T_2_*-weighted MRI acquisitions^26^, its tissue type independence except for adipose tissues^27,28^, and its linearity from 20 to 80 °C^28,29^. It has already been used for a wide range of applications including the monitoring of laser^30^, high intensity focused ultrasound^31^, and RF ablations^9,22^. In clinical applications, the gradient echo (GRE) sequence is widely used for MRTI due to its robustness to magnetic field inhomogeneity artifacts and its ease of use. On static organs, this sequence usually produces temperature maps with an in-plane spatial resolution around 1 mm^2^ and a temporal resolution around 3 s^20,32^.

Real-time MRI guidance during cardiac electro-physiology procedures remains challenging with a number of obstacles preventing its wide clinical adoption^20^. The main ones are associated to the magnetic environment and the need for dedicated hardware including MR-compatible ECG 12-lead monitoring and defibrillators, ablation catheters and software including a real-time communication protocol together with a catheter navigation solution, and real-time image fusion of electro-physiology and MRI information. The inherent complexity of the MRI protocol is also an issue: the limited access to the patient due to the MRI magnet and the presence of the cardiac receiver coils hamper fast and easy defibrillation and sequence parameterization, adjustment, acquisition, and reconstruction workflows need to be simplified to be clinically viable. The use of MR temperature mapping during such critical medical procedures requires a push-button solution for either the physician or the MRI operator. A high level of accuracy but also most importantly reproducibility and repeatability under different physiological conditions is required for MRTI. In early work, monitoring of cardiac ablation was performed with a cardiac and respiratory-gated GRE sequence leading to a temporal resolution of 10 s^9^. This poor temporal resolution motivates the use of ultrafast acquisition sequences such as ECG-triggered gradient echo-Echo Planar Imaging (GRE-EPI) to increase the temporal resolution at the heart rate (approximately 1 s) via single-shot acquisitions^19,22,33–35^. However, the use of GRE-EPI increases in turn the sensitivity to magnetic field inhomogeneity artifacts due to the intrinsic long echo time and low bandwidth in the EPI phase-encoded dimension and decreases the image SNR due to high readout bandwidth.

This proof-of-concept study investigates the use of a non-gated radial golden angle GRE sequence to perform free-breathing continuous cardiac thermometry during RF ablation at high temporal resolution (< 1 s) with reduced distortions as compared to GRE-EPI. The aims of our study were: (1) to automatically localize the catheter in the 2D acquisition plane to allow intra-scan 2D rigid motion correction. The catheter localization was achieved via the signal of its embedded micro-coils which can be localized with two projections^36^ and thus a temporal resolution of two repetition times (≈ 50 ms) while simultaneously acquiring thermometry data with the other coils. The intra-scan 2D rigid motion correction of each projection enables continuous acquisition even if post-acquisition ECG gating limiting out-of-plane motion can also be performed. (2) To accelerate the temporal resolution of temperature maps up to 17× using a direct estimation of the temperature from undersampled k-space data^37^. This was performed by direct fitting of an image model to the k-space data and combined with a hybrid multi-baseline referenceless treatment of the image model correcting for susceptibility induced phase variation due to organ motion and background B_0_ drift^38^. (3) To demonstrate the potential of the method on a moving Agar gel phantom and in vivo in two sheep hearts during MR-guided RF ablations.

## Materials and methods

### Phantom experiments

The Agar-gel phantom experiments were performed with a radiofrequency ablation (RFA) catheter inserted in approximately one liter of 3% Agar-gel in a cylindrical container. The entire phantom (gel and catheter) was set into motion by a pneumatic trolley switching between two positions 26 mm apart at a frequency of 0.33 Hz to roughly simulate respiratory motion.

### In vivo experiment

The in vivo experiment was approved by the ethics committee of Bordeaux University in France and performed in two adult sheep (≈ 50 kg) with one ablation for each. All methods were carried out in accordance with the European directive 2010/63/UE on the protection of animals used for scientific purposes. The study is reported in accordance with ARRIVE guidelines. Each animal was sedated by intramuscular injection of Ketamine (10–20 mg/kg), acepromazine (0.1 mg/kg) and Buprénorphine (9 μg/kg) and anesthetized by an intravenous injection of Propofol (1–2 mg/kg). The animal was intubated after induction of anesthesia, positioned supine in the MRI scanner and ventilated using an MR-compatible ventilator (Aestivia, General Electric, Fairfield, CT, U.S.A.) at 15 breaths per minute (0.25 Hz). Anesthesia was maintained during the whole experiment by continuous breathing of isofluorane (1.5–3%) in a mixture air/oxygen 50/50. Cardiac rhythm and intra-arterial pressure were monitored during the entire experiments (Carescape, General Electric, Fairfield, CT, U.S.A.).

### Radiofrequency ablation device, catheter navigation, and ablation procedure

An MR compatible RFA catheter (Vision-MR Ablation Catheter, Imricor Medical Systems, Burnsville, MN, USA) with two embedded micro-coils (proximal and distal) was used to perform the ablation. The catheter was connected to a clinical RF cardiac ablation generator (Irvine Biomedical, Inc, 1500T11) located inside the Faraday cage, using the Advantage MR system (Imricor Medical Systems, Burnsville, MN, USA).

The RFA catheter was inserted in the femoral artery and navigated through the left ventricle under MRI guidance using active tracking via the prototype Monte Carlo platform (Siemens Healthcare, Erlangen, Germany). A dedicated communication protocol (Scanner remote control, Siemens Healthcare, Erlangen, Germany) was used to transfer either the catheter localization and/or navigation images in real-time to the platform.

The catheter was connected to the RF generator to deliver RF energy at its tip electrode and an adhesive return electrode (MONOPlate, ERBE, Tübingen, Germany) was positioned on the animal’s skin. RF powers of 10 W for the Agar-gel phantom experiments and 30 W for the in vivo experiments were delivered for 60 s each.

### MRI data acquisition

All acquisitions were performed on a 1.5 T Aera MRI system (Siemens Healthcare, Erlangen, Germany) equipped with spine and body coils with 32 and 18 elements, respectively. The radial golden angle (GA) acquisitions were performed with a gradient echo sequence (GRE) without respiratory and ECG triggering. The acquisition parameters were: 220 × 220 mm^2^ Field of View (FoV); 512 readout points accounting for an oversampling factor of 2; 3 mm-thick slice; 250 Hz/pixel readout bandwidth; 20 ms echo time (TE); 24.14 ms repetition time (TR); flip angle (FA) optimized at 15° for the 3%-Agar gel and 10° for the in vivo experiment.

Prior to ablation, a pre-scan of the radial GA GRE sequence was run for a few seconds to ensure motion detection via the micro coils’ signals and localization of the catheter tip in the acquisition slice. A magnitude image was reconstructed by NUFFT using gpuNUFFT^39^ on an external computer in Matlab R2018b after TCP-IP transfer of the raw data using the Gadgetron Framework^40^. Following this step, the GRE sequence was run for 8 min to acquire 20,000 radial projections to monitor the ablation procedure. The in vivo ablation presented in the main text was performed with the slice positioned in a sagittal orientation to minimize the out-of-plane motion due to respiration and the second ablation presented in Supporting Information was monitored in a short-axis orientation. Reconstruction of the temperature maps was performed retrospectively after the acquisition.

The gradient echo-echo planar imaging (GE-EPI) phantom experiment was performed with the same 220 × 220 mm^2^ FoV; 256 readout points including 2× oversampling and 128 phase encode steps with 6/8 Partial Fourier; one 3 mm-thick slice; 1502 Hz/pixel readout bandwidth; 20 ms TE; 1200 ms TR; 90° FA. The matrix size of 128 and the acceleration factor of 2 with GRAPPA reconstruction led to an equivalent inter-echo delay of 0.77 ms and a bandwidth of 10 Hz/pixel in the phase-encoded dimension. The use of factor-two zero filling led to a reconstructed in-plane pixel size of 0.86 × 0.86 mm^2^.

### Data processing

The datasets were converted to ISMRMRD format^41^ and a vendor-neutral processing was implemented in Matlab R2018a (Mathworks, Natick, MA) on a server with 40 CPU cores. The overall data processing is presented by a flowchart in Supporting Information Fig. 1 and detailed in the following section.

### Motion detection

First, the k-space data of the two micro-coils (proximal and distal) embedded in the catheter were separated from the other receiving channels. The Fourier transformation of these micro-coils’ signals gives a zero profile with only one or two close peaks depending on the micro-coil orientation along the projection^42^ as shown in Supporting Information Fig. 2 panels A1, A2, B1 and, B2. This spatial profile is then smoothed by a Gaussian filter to allow a robust localization of the barycenter of the peak even in the case of a split peak. The relative orientation (111.25°) of two consecutive projections enables a 2D Radon transformation to obtain a 2D map of the acquisition plane with one high-intensity spot at the location of each of the micro-coils. Following the position of these high intensity spots allows quantitative 2D catheter motion curves to be extracted. However, the spot position is slightly biased by the projection angle and this bias has to be removed by fitting and subtracting the position over the first projection angle. This fit is illustrated in Supporting Information Fig. 3. The out-of-plane motion is qualitatively assessed by the intensity of the micro coil signal, which decreases when the catheter leaves the acquisition slice. This method^36^ captures the positions of the two catheter micro-coils using only two sequential projections and thus with a temporal resolution of two TR (≈ 50 ms) enabling 2D intra-scan motion correction. A medium-frequency Gaussian filtering with a − 3 dB cut-off frequency of 0.883 Hz is used to filter out the spike artifacts from the raw signal. The respiratory component of the motion curves is extracted using low-frequency Gaussian filtering with a cut-off frequency of 0.377 Hz. The ECG component can also be retrieved by subtracting the respiratory component from the filtered motion.

### Motion correction

The k-space data of the imaging channels were processed with pre-whitening and PCA coil compression with a variance threshold of 90%^43^. Then, each projection was motion-corrected in 2D with a phase shift in the k-space domain as a function of its orientation and the position of the catheter determined previously according to:

$${K}_{corr}=K{\cdot e}^{i2\pi \cdot \left(X-{X}_{mean}\right)\cdot cos\left({\rho }_{R}\right)+1i\pi \cdot \left(Y-{Y}_{mean}\right)\cdot sin\left({\rho }_{R}\right)}.$$

With K the readout, $\text{X}$, and $Y$ the corresponding catheter micro coil positions along the X and Y axes, ${X}_{mean}$ and ${Y}_{mean}$ their mean positions along the X and Y axes, and ${\rho }_{R}$ the projection angle to the X direction.

### Temperature calculation

For each ablation, a learning phase preceding the ablation was used to create an image library, and additional projections were acquired to visually assess temperature stability before the ablation. In the phantom experiment, the learning phase lasted 50 s (2000 spokes) followed by 40 s stability assessment before RF heating. In the in vivo experiment, the learning phase duration was 75 s (3000 spokes) followed by 45 s stability assessment. The image library allows correcting for susceptibility induced phase variations according to the model proposed by Grissom et al.^38^ In this model, the signal at image voxel *j* is defined according to:

$${y}_{j}= \left(\sum_{b=1}^{{N}_{b}}{x}_{b,j}{w}_{b}\right){e}^{i\left({\left\{Ac\right\}}_{j}+{\theta }_{j}\right)}+{\varepsilon }_{j},$$

where ${\left\{{x}_{b}\right\}}_{b=1}^{{N}_{b}}$ are complex-valued library images acquired before heating, ${w}_{b}$ are the baseline image weights, *A* is a matrix of low order polynomial basis function, *c* is a polynomial coefficient vector, $\theta$ is the temperature-induced phase shift and ε is complex Gaussian noise.

For the phantom experiment, a library of 10 images with 180 projections each was created based on the X motion curve. For the in vivo experiment, a library of 24 images corresponding to three cardiac and eight respiratory phases were created based on the respiratory and cardiac motion detected by the catheter micro-coils. This number of cardiac and respiratory phases was found optimal to minimize the susceptibility phase variations (data not shown) while maintaining a sufficient number of spokes per library images comprising between 80 and 172 spokes. For all the experiments a 6th order polynomial was used to create the *A* basis function correcting for B_0_ drift.

The temperature maps were obtained using the method of Gaur et al.^37^ which fits a constrained model directly to undersampled k-space data without an image reconstruction step according to:

$${y}_{j}=\sum_{j=1}^{{N}_{s}}{e}^{l{\overrightarrow{k}}_{i}\cdot {\overrightarrow{x}}_{j}} \left(\sum_{I=1}^{{N}_{b}}{b}_{i,j}{w}_{i}\right){e}^{i\left({\left\{Ac\right\}}_{j}+{\theta }_{j}\right)}+{\varepsilon }_{i},$$

where y_j_ is one k-space data sample, i = 1 to N_k_ indexes the acquired samples, N_s_ is the number of image voxels, k_i_ is the k-space location of sample i, and ${\left\{{b}_{i}\right\}}_{i=1}^{{N}_{b}}$ are the complex baseline library images. The estimation of $\theta$ involves fitting the k-space data with two regularization parameters: λ controlling the sparsity of $\theta$ and β the regularization parameter of a second-order finite differentiating spatial roughness penalty^37,44^.

The performance of this method was assessed for different temporal and spatial resolutions. Temperature maps were reconstructed with 20, 30, and 40 projections leading to temporal resolutions of: 0.48, 0.72, and 0.97 s, respectively, at three in-plane spatial resolutions of 1 × 1 mm^2^, 2 × 2 mm^2^ and 3 × 3 mm^2^ obtained by truncation of the k-space readout. This set of parameters enabled model performance evaluation with acceleration factor ranging from 2.88 to 17.28. The uncertainty of the temperature measurements was computed by the mean and standard deviation of its temporal standard deviation in non-heated regions-of-interest (ROIs) after the training period and before heating.

The regularization parameter β was kept constant while λ was optimized for each spatial resolution by progressively decreasing its value as long as the calculated temperature remains constant allowing accurate temperature determination while minimizing temperature uncertainty. Finally, the temperature maps were filtered temporally using a low-pass Butterworth filter with a cutoff frequency of 0.14 Hz^33^.

The vendor neutral Matlab® processing code is available at: https://github.com/maximeYon/Continuous_Radial_Moco_Thermo. The phantom and in vivo datasets are available at: 10.5281/zenodo.4906122.

## Results

### Comparison of the robustness of radial GRE and GRE-EPI

**Figure 1:**
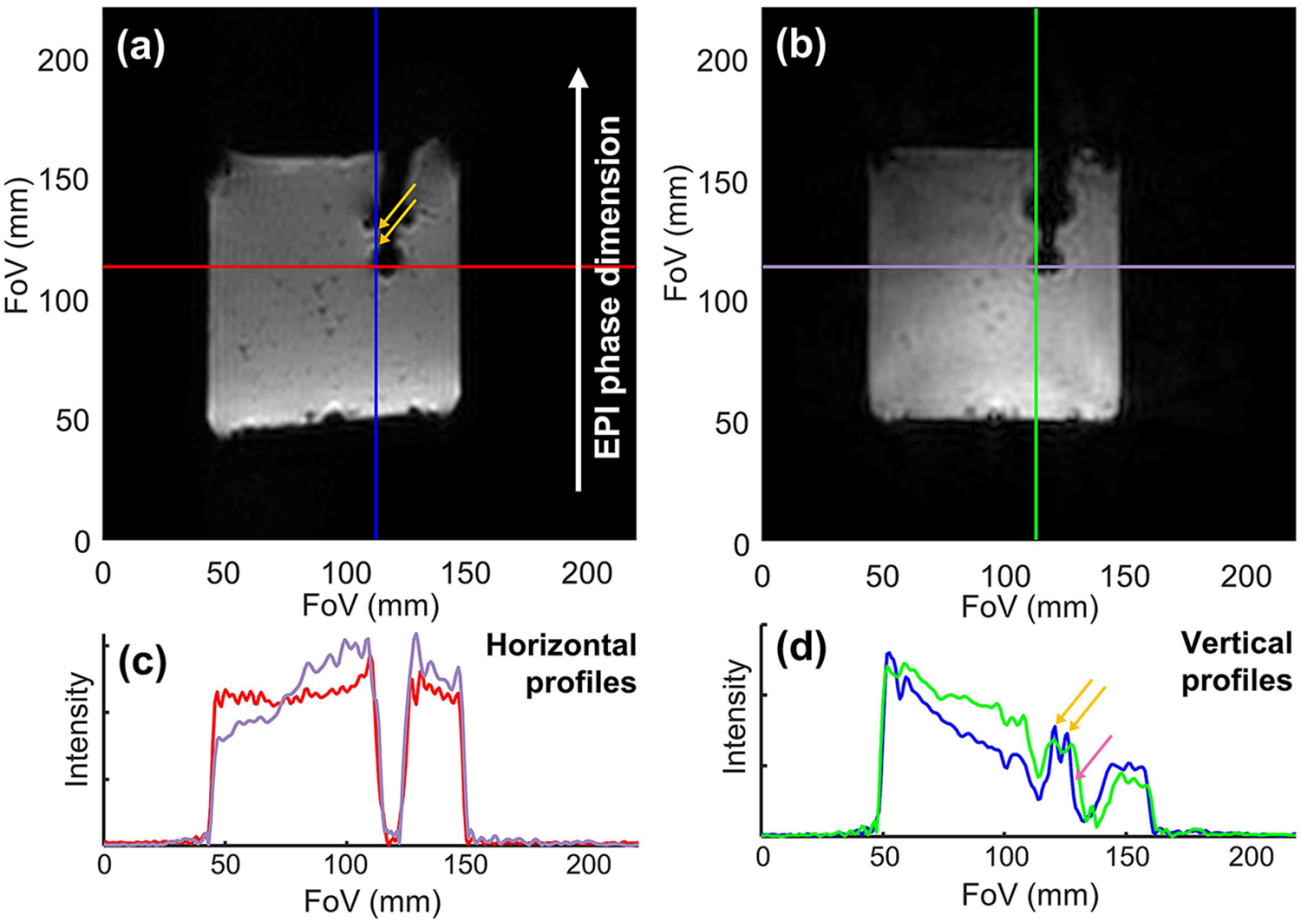
Comparison of the magnitude images and profiles obtained with GRE‐EPI and GRE-radial sequences on a MR compatible catheter inserted in an Agar gel phantom. (**a**) GE-EPI image. (**b**) GRE-radial golden angle image. Both images were acquired with an identical TE, spatial resolution and shim conditions. (**c**) Horizontal profiles corresponding to the locations of the red and purple lines in the EPI and GRE images respectively. (**d**) Vertical profiles corresponding to the locations of the blue and green lines in the EPI and GRE images respectively. The orange arrows exhibit the B_0_ inhomogeneity artifacts appearing as signal super-intensity especially present in the EPI image and the pink arrow exhibits the B_0_ inhomogeneity artifacts appearing as a shift between the EPI and GRE image and equal to 3.4 mm.

Figure 1 shows a comparison between a GRE-EPI image and a radial golden angle GRE image of an MR compatible RFA catheter inserted in a 3% Agar gel phantom and acquired with an identical TE of 20 ms, identical shim conditions, and identical spatial resolutions. The GRE-EPI image exhibits obvious geometric distortions due to B_0_ inhomogeneity in the vertical (phase encoding) dimension, which are especially severe close to the interface between the gel and the catheter tip and micro-coils. A comparison of the vertical and horizontal intensity profiles (blue versus green and red versus purple, respectively) is shown in panel c and highlights the good correspondence of the horizontal profiles while the vertical profiles show many more differences. The orange arrow indicates signal pile-up in the GRE-EPI image due to magnetic field inhomogeneity and the pink arrow indicates a shift of 3.4 mm between the EPI and GRE images. However, the round black spot localized at the catheter tip and due to intravoxel dephasing appears to be of the same diameter as shown by the nearly perfect superposition of the purple and red profiles.

### Phantom catheter localization and motion correction

**Figure 2:**
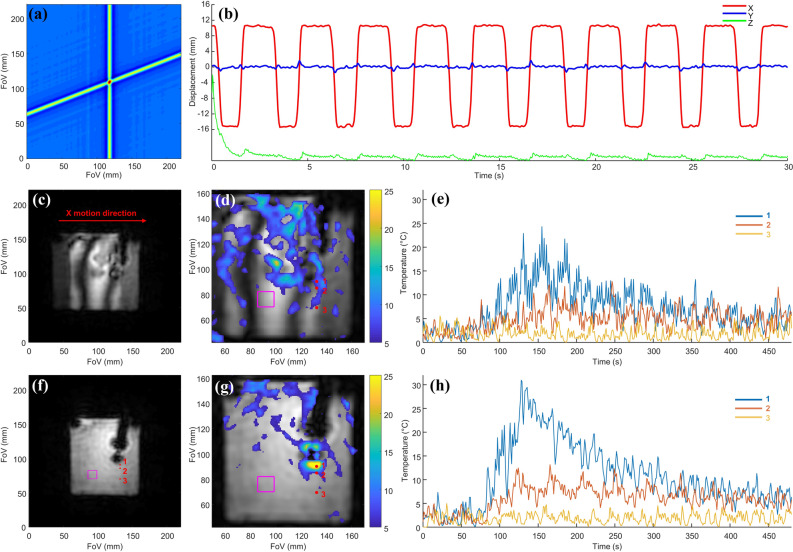
Motion detection and effect of the motion correction on magnitude images and temperature maps on a moving Agar gel phantom: (**a**) magnitude image obtained by 2D radon transform of two projections and used for localizing each of the catheter micro-coils. (**b**) Motion curves of the distal micro-coil of the RFA catheter inserted in the moving Agar phantom. The X (red) and Y (blue) curves describe the in-plane motion quantitatively while the Z (green) curve shows qualitatively the variation of the micro-coil signal intensity. (**c,f**) show magnitude images reconstructed by NUFFT with an in-plane resolution of 2 × 2 mm^2^, all the projections (20,000) without and with rigid 2D in-plane motion correction respectively. (**d,g**) Show temperature maps obtained at t = 135 s with 40 projections without and with motion correction, respectively. Temperature values below 5 degrees are set to transparent. (**e,h**) Show three temperature curves obtained in points 1, 2, and 3 without and with motion correction, respectively. The temperature standard deviations obtained in the purple box area after the training period (from t = 50 s) were 1 °C and 1.5 °C with and without motion correction, respectively.

Figure 2a shows an example 2D Radon transform from two consecutive projections of the distal (closest to the tip) micro coil’s profiles. The position of the micro-coil was determined quantitatively in 2D as the position with maximum intensity. The motion curves of the catheter micro coil placed in the 3% Agar gel phantom and positioned on a pneumatic trolley are shown in Fig. 2b. The X and Y curves describe quantitatively the motion of the trolley switching between two positions distant from 26 mm along the X-axis with a frequency of 0.33 Hz. The Z curve provides a qualitative idea of the out-of-plane motion based on the intensity of the catheter signal in the selected slice. The exponential decay at the beginning of this curve is due to the establishment of the magnetization steady state. The temporal resolution of this curve is 48.28 ms and corresponds to the acquisition of two consecutive projections. The uncertainty of the in-plane position was quantified by the standard deviation of the motion curves of a static object and is 0.3 pixels corresponding to 0.26 mm (data not shown). The result of the intra-scan motion correction can be appreciated in Fig. 2c,f which display the magnitude images reconstructed by NUFFT with an in-plane resolution of 2 × 2 mm^2^ using all the 20,000 projections, without and with motion correction respectively.

Figure 2e,h show three temperature curves representative of three heating intensities obtained at different spatial locations (points 1, 2, and 3) without and with motion correction, respectively. In the absence of motion correction, the hot spot is spread due to the sequential motion of the phantom (panel 2d) and the corresponding temperature evolution curves show large oscillations. Using the motion correction method, a more realistic spatial distribution of the temperature is recovered with only one main heating spot. The temperature curves show drastically reduced oscillations and a higher maximum temperature. The standard deviation of the background temperature measured in a ROI without heating shown with a purple square on the images in Fig. 2d,g, is also decreased from 1.5 to 1 °C by the motion correction.

### Acceleration, temporal resolution, spatial resolution, and uncertainty of phantom temperature measurement

**Figure 3:**
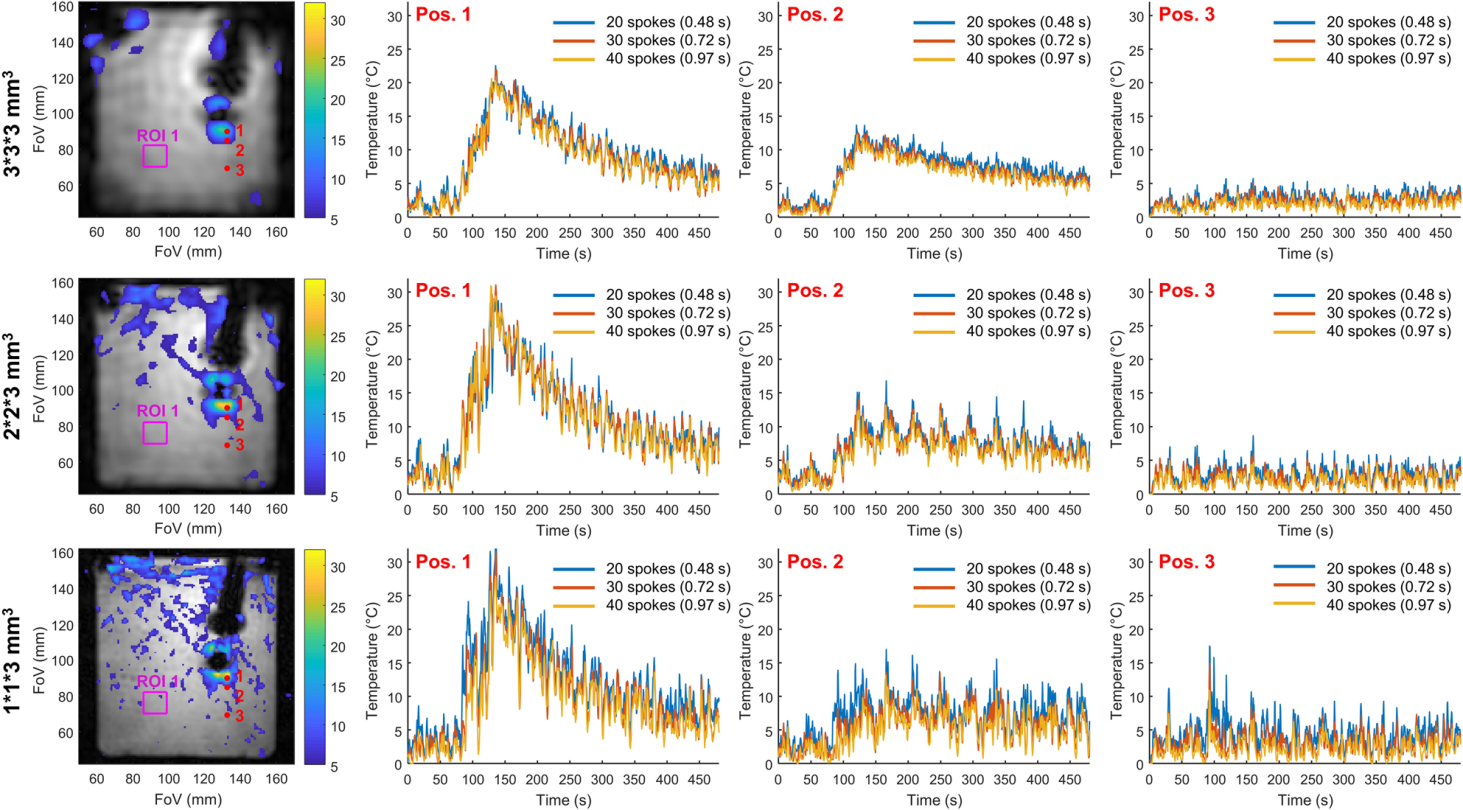
Influence of the temporal and spatial resolutions on temperature maps of a moving Agar gel phantom. Left column: temperature maps obtained with 40 projections a time = 135 s with an in-plane resolution of 1 × 1 mm^2^, 2 × 2 mm^2^ and 3 × 3 mm^2^ from top to bottom and a 3 mm slice thickness. The regularization parameters are β = 0.05 and λ = 0.1, 0.2 and 0.5 for the resolution 1 × 1, 2 × 2 and 3 × 3, respectively. The temperature curves are presented at three spatial locations identified by the red points 1, 2, 3. The three curves: blue, red, and yellow show the results at three temporal resolutions: 0.48, 0.72, and 0.97 s Each curve has been temporally filtered with a Butterworth filter with a cut of frequency of 0.14 Hz. The acceleration, the mean standard deviation in the purple ROI are given in Table 1.

A comparison between three in-plane resolutions: 1 × 1, 2 × 2, and 3 × 3 mm^2^ for three different temporal resolutions based on the same dataset is shown in Fig. 3. The spatial resolution of the thermometry map was decreased by truncation of the k-space readout, decreasing, in turn, the number of projections required to sample the entire k-space. The temporal resolution was tuned to 0.48, 0.72, and 0.97 s by adjusting the number of projections used to compute the temperature maps to 20, 30, and 40, respectively. At a defined update rate of thermometry images the acceleration factor decreases with the decrease in spatial resolution. The temperature curves corresponding to the three temporal resolutions agree well, validating the value of the regularization parameters (λ and β) given in Table 1. The mean standard deviation values in the ROI far from the heating zone are summarized in Table 1. The important oscillations of the temperature curve, especially present in position 2 at high spatial resolution are due to incomplete phase compensation of the motion by the libraries, which did not contain enough images to compensate for the very fast switching motion of the trolley.

**Table 1:** Accelerations, regularization parameters, calculation times required to reconstruct all the temperature maps of the 8 min in vivo acquisition, and mean standard deviations in the purple ROIs after the training period for the Agar-gel and the in vivo experiments.

Spatial resolution	Temporal resolution			
	0.97 s Number of projections: 40	0.72 s Number of projections: 30	0.48 s Number of projections: 20	Regularization parameters
3 × 3 × 3 mm^3^ Matrix size: 74 × 74 × 1	Acceleration: 2.88 calc. time: 12 min Agar-gel std: 0.6 ± 0.1 °C In vivo ROI 1 std: 1.3 ± 0.2 °C In vivo ROI 2 std: 0.2 ± 0.1 °C	Acceleration: 3.84 calc. time: 15 min Agar-gel std:0.7 ± 0.1 °C In vivo ROI 1 std: 1.2 ± 0.2 °C In vivo ROI 2 std: 0.3 ± 0.1 °C	Acceleration: 5.76 calc. time: 20 min Agar-gel std:0.8 ± 0.1 °C In vivo ROI 1 std: 1.2 ± 0.2 °C In vivo ROI 2 std: 0.4 ± 0.1 °C	λ = 0.5 β = 0.05
2 × 2 × 3 mm^3^ Matrix size: 110 × 110 × 1	Acceleration: 4.32 calc. time: 15 min Agar-gel std:1.0 ± 0.1 °C In vivo ROI 1 std: 1.5 ± 0.1 °C In vivo ROI 2 std: 0.5 ± 0.1 °C	Acceleration: 5.76 calc. time: 17 min Agar-gel std:1.1 ± 0.1 °C In vivo ROI 1 std: 1.6 ± 0.1 °C In vivo ROI 2 std: 0.7 ± 0.1 °C	Acceleration: 8.64 calc. time: 24 min Agar-gel std:1.2 ± 0.1 °C In vivo ROI 1 std: 1.6 ± 0.1 °C In vivo ROI 2 std: 0.9 ± 0.1 °C	λ = 0.2 β = 0.05
1 × 1 × 3 mm^3^ Matrix size: 220 × 220 × 1	Acceleration: 8.64 calc. time: 21 min Agar-gel std:1.2 ± 0.2 °C In vivo ROI 1 std:1.7 ± 0.1 °C In vivo ROI 2 std: 1.1 ± 0.2 °C	Acceleration: 11.52 calc. time: 28 min Agar-gel std:1.4 ± 0.2 °C In vivo ROI 1 std:1.8 ± 0.1 °C In vivo ROI 2 std: 1.3 ± 0.2 °C	Acceleration: 17.28 calc. time: 44 min Agar-gel std:1.7 ± 0.17 °C In vivo ROI 1 std:1.8 ± 0.2 °C In vivo ROI 2 std: 1.5 ± 0.2 °C	λ = 0.1 β = 0.05

### In vivo experiment

**Figure 4:**
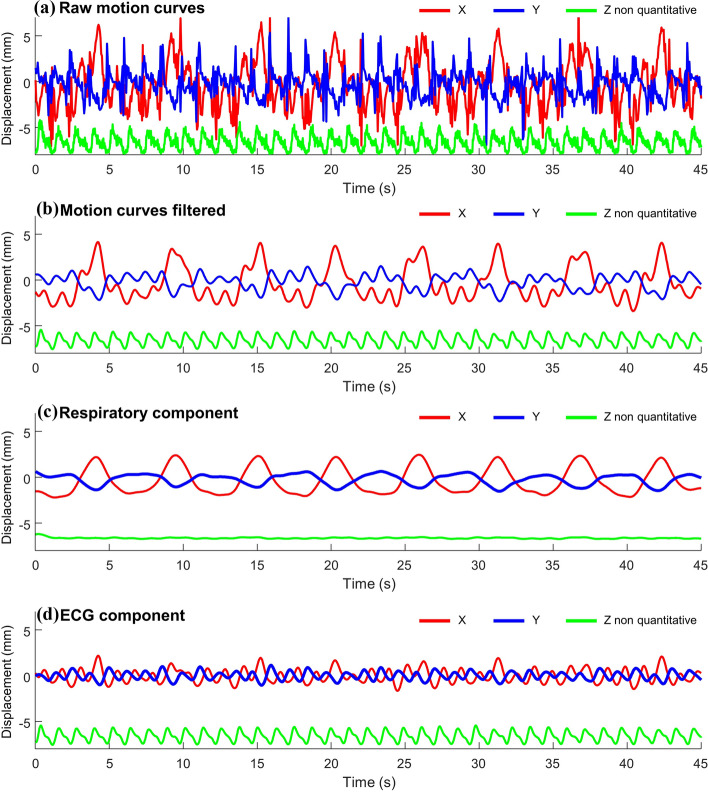
Motion curves obtained by following the catheter micro coils by Radon transformation of two consecutive radial projections with the catheter in contact with the myocardium of the left ventricle. The signals have a temporal resolution of two TR (≈50 ms) and the two (proximal and distal) coil signals have been averaged. (**a**) Motion curves without any temporal filtering. (**b**) Motion curves filtered by medium frequency Gaussian filter with a cut of frequency at 0.883 Hz. (**c**) Respiratory component of the motion curves obtained by Gaussian low-pass temporal filtering with a cut-off frequency of 0.377 Hz. (**d**) ECG component of the motion curve obtained by subtracting the respiratory component from the signal filtered by medium-frequency Gaussian. In all cases, the in-plane motion curves are mean-centered to 0 while the Z intensity curve is artificially displayed with an offset of − 6.6 mm for the sake of clarity.

Figure 4 shows the mean motion curves of the two catheter micro-coils obtained during the in vivo acquisition with a temporal resolution of ≈ 50 ms. Figure 4a plots the raw motion curves: quantitative for the in-plane (X and Y) motion and qualitative for the Z direction. The raw in-plane motion is difficult to interpret without filtering due to the presence of spikes even if some features of the respiratory motion can be distinguished, especially in the X-direction. The micro-coil signal intensity, labeled as “Z intensity”, is much less noisy and exhibits a regular pattern whose frequency (0.825 Hz) matches the expected ECG frequency.

**Figure 5:**
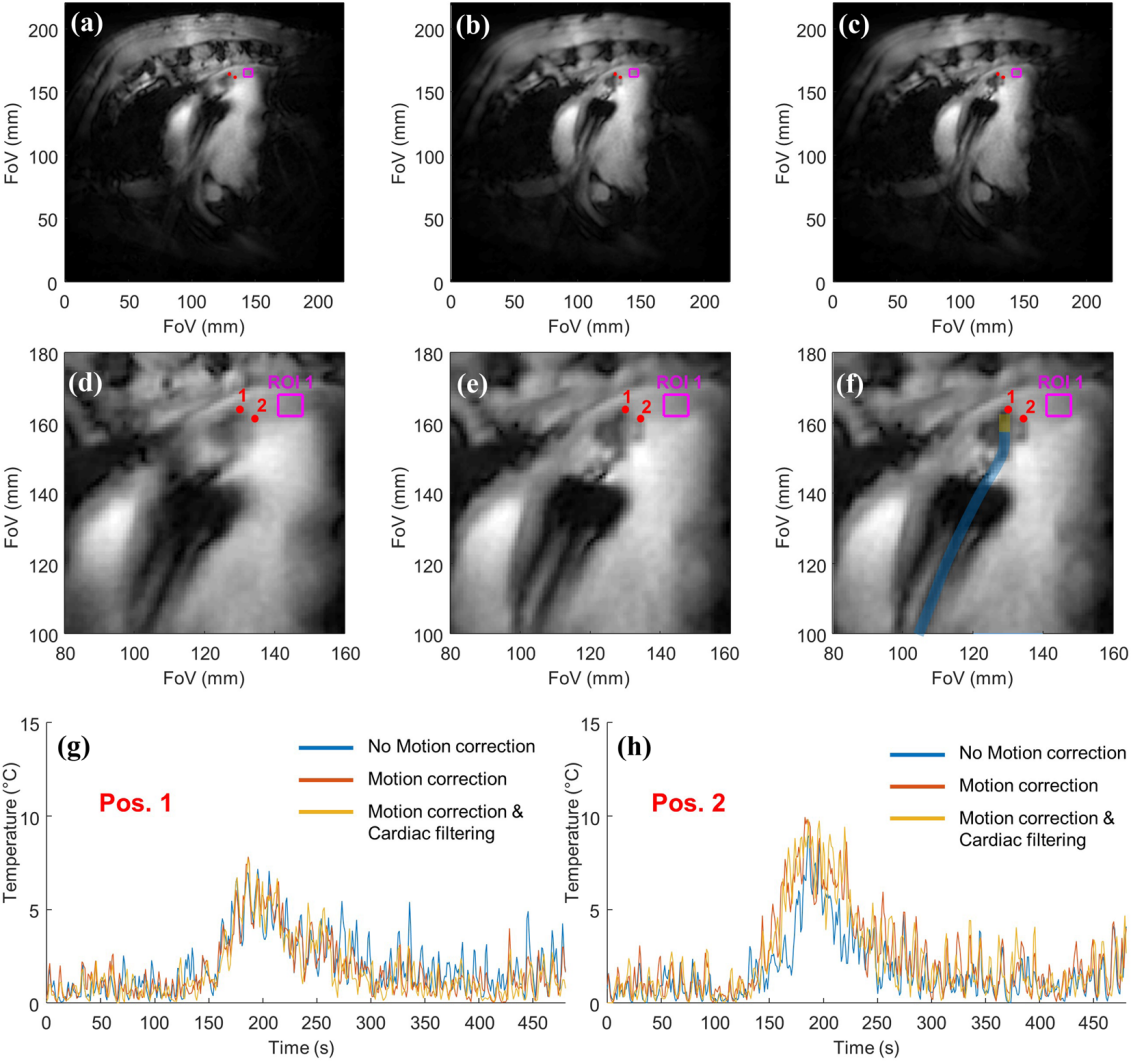
Radial GRE magnitude images and temperature evolution curves acquired in sagittal orientation during RF ablation. Magnitude images in full FoV (first row) and zoomed view (second row) reconstructed by NUFFT with the projections acquired during the entire 8 min acquisition with a resolution of 0.86 × 0.86 × 3 mm^3^. (**a,d**) Without motion correction; (**b,e**) with motion correction based on total filtered motion curves; (**c,f**) reconstructed with motion correction and selection of only 70% of the projection based on cardiac cycle. A schematic representation of the approximate position of the catheter is shown in (**f**). (**g,h**) Temperature curves at points 1 and 2, respectively. The mean standard deviations in the square ROI were 1.6, 1.5, and 1.6 °C for the temperature obtained without motion correction, with motion correction, and with motion correction and cardiac phase filtering, respectively.

A medium frequency Gaussian filtering (cut of frequency = 0.883 Hz) allows to filter out the spike present in the raw signal and to obtain smooth motion curves shown in Fig. 4b. The respiratory component of each of the motion curves was retrieved by applying a low-frequency Gaussian filtering (cut of frequency = 0.377 Hz) and is displayed in Fig. 4c. The in-plane respiratory component of the motion is apparent and has a maximum amplitude of 4.5 mm in X: vertical/dorsal–ventral direction and 2.12 mm in Y: the horizontal/antero-posterior direction. The Z intensity does not show a meaningful respiratory component suggesting the absence of out-of-plane motion due to the respiration in this acquisition. The ECG component was retrieved by the subtraction of the respiratory component from the filtered motion and is shown in Fig. 4d. The maximum amplitude of this component was 1.92 mm in X and 1.52 mm in Y. The periodic overshoots in the X direction are due to incomplete filtering of the respiratory component with the low frequency filter. The motion correction of the overall filtered motion (respiratory plus ECG components) lead to the best motion correction and was used to obtain the corrected image in Fig. 5b,e. Superimposition between the filtered motion curve and the raw motion curve is presented in Supporting Information Fig. 4. The motion curves obtained in the second in vivo experiment in the short axis are shown in Supporting Information Fig. 6. These curves show similar behavior except for the Z intensity signal, which has a respiratory component due to the short axis orientation of the acquisition slice. The motion curves are shown for the entire experiment in Supporting Information Figs. 7 and 8 for the sagittal and short-axis orientations, respectively. Important perturbations of the cardiac rhythm and of the catheter motion amplitude are visible during the ablation: from 120 to 180 s and during the 70 s following the end of the RF energy delivery.

Figure 5 shows the magnitude images obtained by NUFFT reconstruction of the 20,000 projections acquired during the 8-min experiment. The first line shows the full FoV and the second line shows a zoomed view of the heart. The images were reconstructed without readout truncation and exhibit an in-plane resolution of 0.86 × 0.86 mm^2^. Three reconstructions are shown: (i) without motion correction (panels a,d), (ii) with rigid 2D motion correction (panels b,e) based on the filtered motion curves displayed in Fig. 4d, (iii) with motion correction and only 70% of the projections (Fig. 5c,g). The projection selection based on the micro-coil signal intensity (Z signal) shown in Fig. 4 in green was set to decrease the out-of-plane motion by discarding the projection acquired during fast Z intensity variations. The selected and unselected zones of the Z-intensity curve are displayed in Supporting Information Fig. 5. The motion correction leads to an increased sharpness of the image close to the catheter and in the interventricular septum. Two temperature curves obtained for the red points 1 and 2 are shown in Fig. 5g,h, respectively. The mean standard deviation of the temperature computed (after the 75 s training period) over the square ROI was 1.6, 1.5, and 1.6 °C without motion correction, with motion correction, and with motion correction and cardiac phase filtering, respectively. Since the selection of the projection does not improve the temperature uncertainty or shape of the heating curve, no selection was performed for the subsequent parameter optimizations. The Fig. 5 is reproduced in Supporting Information Fig. 9 for the second ablation in short-axis orientation.

**Figure 6:**
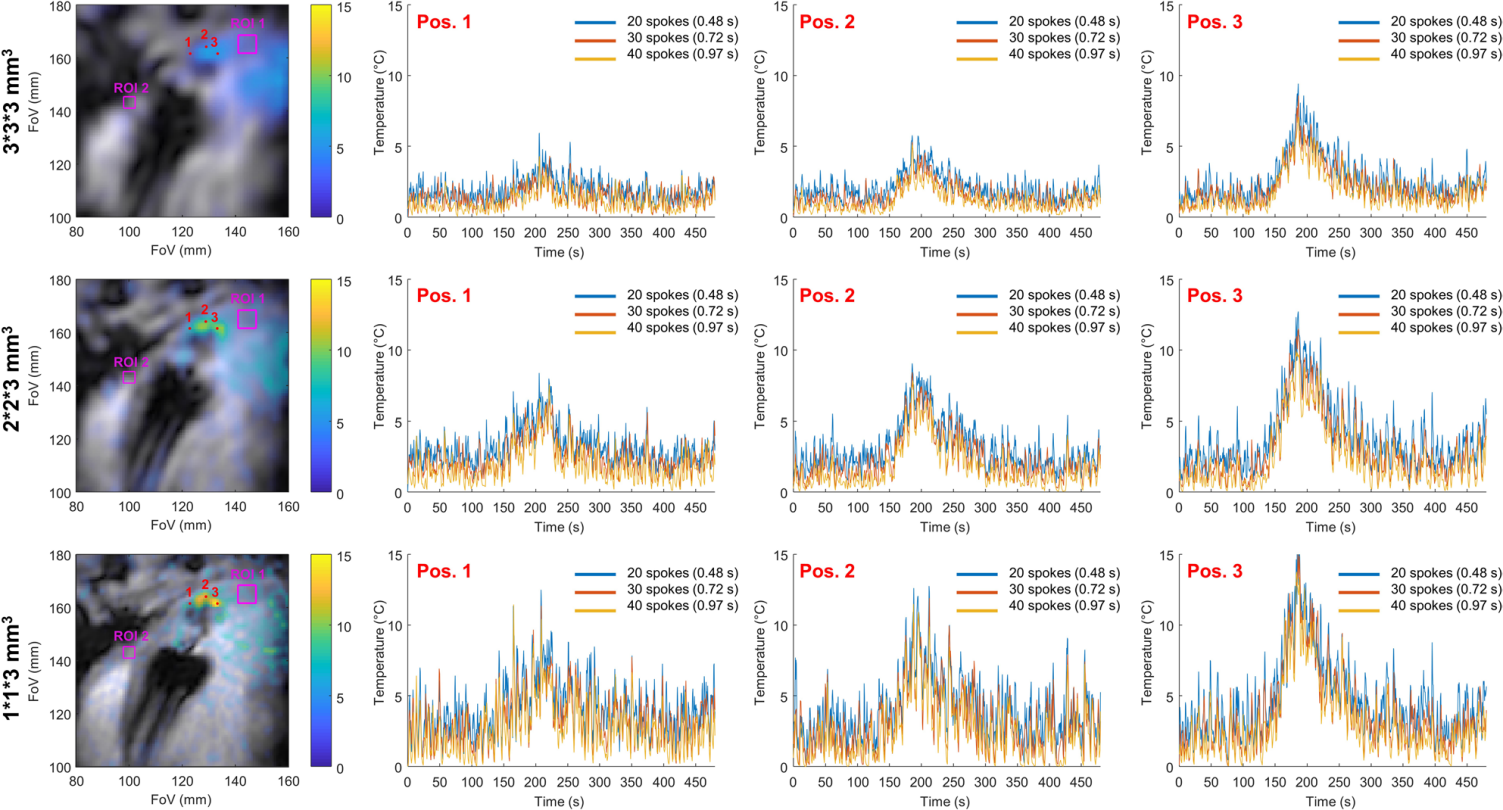
Influence of temporal and spatial resolution on temperature maps in the in vivo experiment. Left column: temperature maps obtained with 40 projections a time = 188 s with in-plan resolutions of 1 × 1 mm^2^, 2 × 2 mm^2^ and 3 × 3 mm^2^ from top to bottom and a 3 mm slice thickness. The regularization parameters are beta = 0.05 and lambda = 0.1, 0.2 and 0.5 for the resolution 1 × 1, 2 × 2 and 3 × 3 mm^2^, respectively. The temperature curves are presented at three spatial locations identified by the red points 1, 2, 3. The three curves: blue, red, and yellow show the results at three temporal resolutions: 0.48, 0.72, and 0.97 s corresponding respectively to the acquisition of 20, 30, and 40 consecutive projections. Each curve has been temporally filtered with a Butterworth filter with a cut of frequency of 0.14 Hz. The accelerations, the mean standard deviations in the ROIs, and the calculation times are given in Table 1.

Figure 6 shows the temperature maps at t = 188 s and the temporal evolution of the temperature in three spatial locations for three in-plane resolutions: 1 × 1, 2 × 2, and 3 × 3 mm^2^ and for three different temporal resolutions: 0.48, 0.72, and 0.97 s. The heating can be followed for each of the spatial resolutions and the curves corresponding to 20, 30, and 40 projections are well superimposable validating the choice of the regularization parameters. The values of the mean standard deviation in the two purple ROIs are given in Table 1. In the ROI #1 zone, the standard deviations were between 1.2 and 1.9 °C while in the second ROI #2 they were between 0.2 and 1.5 °C. Close to the apex, in ROI #1, the temperature fluctuations were dominated by residual susceptibility-induced phase shifts due to the respiratory motion. This explains why the standard deviation of the temperature was not substantially dependent on the spatial and the temporal resolutions and remains high. In ROI #2, the noise is the main contributor to temperature uncertainty and the standard deviation values are lower but increase more with the increase in spatial and temporal resolution. The improved spatial resolution also leads to a decrease in partial volume effect increasing the maximal temperature in the small heating spots close to the catheter. This figure is reproduced in Supporting Information Fig. 10 for the second ablation in short-axis orientation.

## Discussion

Previous studies^19,22,33,35^ have shown that ECG-triggered GRE-EPI with free-breathing can be used for the monitoring of lesion formation during RFA. There are three main limitations to this approach. First, the trigger of the acquisition depends on cardiac rhythm detection. In practice, any modification of the ECG pattern during the ablation is likely to modify the rate or interrupt the acquisition of temperature images during this critical period. Second, the acquisition parameters of the GRE-EPI sequence must be carefully balanced between the acquisition train length, the use of a small FOV leading to inherent aliasing in the phase-encoded direction and the high spatial resolution required to image the lesion whose size is usually in the order of 3 to 10 mm. Finally, B_0_ inhomogeneity artifacts remain an important drawback, especially when a contact-heating device such as an RF catheter is used. Indeed, the susceptibility differences at the device interface lead to intra-voxel dephasing creating a blind spot artifact and geometric distortions of the temperature map^19^ as shown in Fig. 1.

In this proof-of-concept study, we investigated the use of an ungated radial golden angle GRE sequence for continuous temperature monitoring in an Agar-gel phantom and in vivo to monitor cardiac RFA. The main findings of this study were that: (1) The sequence is easy to set up without many compromise between imaging parameter and echo time, it is also not subject to aliasing artifacts and reduces susceptibility induced distortions at the cost of spatial coverage, which is lower in the third (slice) dimension compared to GRE-EPI. (2) Radon transformation of the micro-coil signal embedded in the catheter allows detecting and correcting in 2D the respiratory and cardiac motion. (3) The method has limited sensitivity to motion due to the robustness of the radial encoding scheme coupled with intra-scan motion correction and allows temperature imaging even during irregular heart contractions. (4) Temperature imaging using highly undersampled radial trajectories combined with direct k-space estimation is feasible and enables temporal resolutions better than 1 s, suitable for monitoring cardiac RF ablations.

The robustness of the method to magnetic field susceptibility artifacts is due to the GRE radial acquisition whose bandwidth is widely adaptive in the two dimensions and can be set to high values (here 250 Hz/pixel) which minimizes distortion. In comparison, the bandwidth of the GRE EPI sequence in the phase-encoded dimension is usually much lower (here 10 Hz/pixel) and highly constrained by the FoV, the spatial resolution and gradient performance. This bandwidth difference has no impact of the size of the intra-voxel dephasing artifact at the tip of the catheter, which remain of similar size in the EPI and radial GRE images. The only way to reduce this artifact would be to decrease the TE at the expense of accuracy of the temperature measurement^45^. This is illustrated in Supporting Information Fig. 11 where the magnitude images and temperature uncertainty are compared for the GRE-EPI and the radial GRE sequence with different TE. In our case, at constant temporal resolution this decrease in temperature accuracy is partially compensated by the acquisition of more projections in the same acquisition time. The TE value can be strongly reduced in GRE without compromise while in EPI the TE is already close to its minimal value and cannot be reduced without compromise on the FoV or spatial resolution. However, the acquisition time of a fully sampled GRE radial image is also much longer than the one of its EPI counterpart, especially for thermometry when the echo time is not set to its minimum value to be in the range of TE ≈ T_2_* leading to optimal thermometry uncertainty^45^. This longer acquisition time increases the sensitivity of the GRE to intra scan motion artifact compared to EPI and decreases the achievable temporal resolution of the image series acquisition used for temperature monitoring to tens of seconds without undersampling. The monitoring of cardiac RFA requires both a high robustness to susceptibility artefact and a high temporal resolution, it is then mandatory to accelerate the acquisition to be able to use a GRE acquisition.

The direct estimation of the temperature from undersampled k-space data^37^ is currently the method providing the highest acceleration (up to 30 in radial acquisition) for MRTI and has already proved its efficiency for in vivo ablation in the brain^37^ and liver^46^. It is combined with hybrid referenceless and multibaseline substraction^38^ allowing to correct the phase variation due to the organ motion. This correction is performed in two steps: first, during the learning phase (before the ablation), incoming data are stored, sorted, and used to create a mathematical library of the susceptibility variations. Then, during the interventional step (the ablation), this library is used to remove the matching background anatomical phase from the calculated temperature map. The number of baseline images was optimized on the resulting temperature uncertainty for each experiment to find the best compromise between the number of physiological (cardiac and respiratory) phases and the number of projections per phase allowing the reconstruction of a meaningful library image. In addition, a smooth low order polynomial phase is also fitted and subtracted to correct for B_0_ drift.

However, this method does not correct for the intra-scan motion of the heating spot, which is unavoidable in cardiac RFA without very restrictive cardiac and respiratory triggering prohibiting high temporal resolution. Indeed, despite the well-established robustness to intra-scan motion artefacts^47^ of the radial GA GRE, intra scan motion still induce blurring in the magnitude and phase images^47^. Various strategies exist for intra-scan motion correction based on phase correlation methods^48^, auto-focusing^49^, repeated acquisition of a navigator^50^, or tracking of an internal device^36^. In cardiac thermometry, the use of long TR combined with the fast-cardiac motion prohibits the use of the two first types of method requiring an image reconstruction step and thus the acquisition of tens of projections limiting the temporal resolution of the motion correction. The navigator and tracking methods allow motion correction with a temporal resolution of one or two TR compatible with real-time thermometry. The tracking of the catheter was chosen due to its simplicity (no additional signal acquisition period or gradients) and its ability to describe the motion in 2D. We then used the available signal of the embedded micro coils to detect the physiological motion, which after Radon transformation of the 1D profiles allows extracting the 2D motion. A 2D rigid motion correction was applied to each projection before MRTI processing. This method originally developed by Rashe et al.^36^ was found to be highly reliable since it works in the phantom and in vivo for both the sagittal and the short axis orientation as shown in Fig. 4 and Supporting Information Fig. 6. It gives quantitative motion curves of the catheter micro coils with high precision (0.3 mm) allowing intra-scan motion correction on each couple of radial projections as well as a clean qualitative assessment of the out-of-plane motion allowing ECG-gating. The efficiency of the motion detection and correction was evaluated in Agar-gel (Fig. 2 panels c,d,f,g) and in vivo (Fig. 5 panels a,b,d,e). For all cases, it induces a sharpening of the magnitude image in the catheter area. The motion correction also improves the shape of the heating curve and increases the maximum temperature detected by avoiding in-plane intra-scan motion of the heating spot. In the phantom experiment (Fig. 2), it allows an increase of approximately 7 °C close to the tip of the electrode due to the freezing of the motion of the heating spot. In Agar-gel, the motion correction also improves the temperature uncertainty: the standard deviation in the ROI decrease from 1.5 without to 1.0 °C with it. In vivo, the effect of the motion correction on the temperature is more subtle: it increases the maximum temperature by approximately 3 °C in both experiment and improves the shape of the heating curve but has no clear benefit on the temperature uncertainty in the ROI far from the heating. This is especially evident in Supporting Information Fig. 9 panels h and h where the motion corrected temperature curve present additional rapid oscillations.

In the two in vivo experiments, the use of cardiac gating with the selection of 70% of the projection slightly improves the sharpness of the magnitude image but also increases the temperature uncertainty measured in the ROIs. This can be explained by the decrease in temporal resolution leading to decreased efficiency of the temporal Butterworth filter. Since no major change in the measured temperature was observed with cardiac gating, it will not be used. It is probable than the effect of the out-of-plane motion is relatively weak due to the important slice thickness.

Temperature maps were successfully reconstructed in a phantom and in vivo acquisitions allowing the visualization of the temperature elevation close to the catheter tip even during RF ablation when the cardiac rhythm is perturbed. The direct estimation of the temperature from undersampled k-space data algorithm succeed to provide temperature maps with acceleration ranging from 2.8 to 17 without penalty other than the increase in temperature uncertainty probably due to the SNR reduction. The regularization parameters β (value 0.05) and λ (values 0.5, 0.2, 0.1) were found stable across experiments and succeed to give superimposable temperature curves for the various temporal resolution. The temperature values for the different spatial resolutions seem to agree, even if this is difficult to assess due to partial volume effects changing with the resolution. The uncertainty of the temperature measurement evolves in an inversely proportional way with the spatial and temporal resolutions for both the phantom and in vivo experiments (Table 1).

The temperature uncertainty (around 2 °C) was in the order of magnitude of previous in vivo studies at similar temporal resolutions using GRE-EPI: 3.6 °C ± 0.9 °C, De Senneville et al.^22^; 0.67 °C ± 0.24 °C, Ozenne et al.^33^; 1.5 °C ± 0.4 °C, Toupin et al.^19^; 1.52 °C ± 0.51 °C.

### Limitations

Several limitations remain on the way to real-time clinical applications. First, the current implementation of the reconstruction algorithm is not yet compatible with real-time reconstruction since the average calculation times are twice longer than the acquisition. This is not a critical issue since solution for faster computing exist. Multi-GPU reconstruction where each incoming frame during the interventional phase is independently reconstructed could be envisioned^51^ as well as the use of faster algorithm based on low resolutions library images^46^.

Classification of the radial projection for the library creation was done with a regular cardiac rhythm while the targeted population is highly susceptible to irregular heart rhythms. Robustness of the phase subtraction to the variations in RR intervals between beats is a second potential issue needing to be evaluated before clinical application. In such cases, classification of beats based on RR interval^34,52^ or QRS morphology^53^ could also be envisioned to sort the projection during the library creation. The fast k-space algorithm^46^ could alleviate this limitation by providing a more robust physiological phase compensation toward outliers.

The temperature uncertainty is approximately three time higher than the one of the state of the art counterpart method based on GRE-EPI^33^. This uncertainty is partially due to the intrinsic lower SNR due to the high undersampling factor in the GRE acquisition but can also be attributed to the increase of noise due to rigid motion correction. This effect is probably due to incoherent artifacts produced by the 2D rigid motion correction on the non or differently moving part of the image. This drawback could be alleviated with the use of a non-rigid motion correction^49,54^.

Spatial coverage of the method is lower in the 3rd dimension compared to multi-slice GRE-EPI where up to 6 slices per heartbeat covering a FOV of 180 × 180 × 30 can be acquired. Echo shifted multislice acquisition^55,56^ could be used to increase the spatial coverage without additional cost in temporal resolution.

The ablation catheter’s tip and micro-coils create large and intense susceptibility artifacts (Figs. 1, 5, 6) prohibiting to image its close vicinity due to intra-voxel dephasing and blurring its surroundings due to localized B0 inhomogeneities. This limits our capacity to get temperature data from the catheter’s closest voxels where the temperature increase is the more important. These artefacts can be diminished by shortening the echo time (at the expense of temperature precision^45^) or by using susceptibility matched catheters^57^. In ventricular ablation, the ability to map tissue temperature close to the catheter is not crucial since the relevant information for the clinician is the extent of the lesion and its transmurality.

The number of in vivo experiments is too low to absolutely demonstrate the clinical utility of the method but the diversity of experimental conditions is sufficient to point out that undersampled radial sequence can be considered as an alternative to GRE-EPI for monitoring cardiac RFA.

## Conclusion

The method combining continuous GA radial acquisition, intra-scan motion correction and direct estimation of temperature from undersampled k-space data was successfully applied in two in vivo ablations at high temporal (less than a second) resolution. This method provides better robustness to image distortion artifact due to magnetic field susceptibility differences compared to its GRE-EPI counterparts at the expense of an intrinsic lower SNR and thus temperature uncertainty^15^. Moreover, the catheter tracking concomitant with the data acquisition allows to measure the motion of the catheter tip exactly in the region of interest for temperature mapping and perform intra-scan 2D rigid motion correction improving even more the robustness of the method toward in plan motion. While being preliminary, the proposed solution alleviates some inherent default of the GRE-EPI for the temperature monitoring of cardiac RFA and could be used as a push-button solution. While this option was not tested here, the embedded micro coils can be used as an active tracking system that automatically moves the slice at the tip of the catheter. This could lead to a relatively simple scenario where orientation and resolution are the only parameters to set up at the beginning of the exam. This could allow MRTI to be used on demand (using a foot sensor) by the clinician without any adjustment during the procedure.

## Supplementary Information

Supplementary Figures.

## Data Availability

The datasets generated and/or analysed during the current study are available at: 10.5281/zenodo.4906122.

## Code availability

The Matlab code used to generate the results of this study is available at: https://github.com/maximeYon/Continuous_Radial_Moco_Thermo.
