# Supplementary material for: Continuous cardiac thermometry via simultaneous catheter tracking and undersampled radial golden angle acquisition for radiofrequency ablation monitoring
Source: Sci Rep. 2022 Mar 7;12:4006. doi: 10.1038/s41598-022-06927-9 (PMC8901729; doi:10.1038/s41598-022-06927-9)
Supplement: Supplementary file 1 — Supplementary Figures. [file 41598_2022_6927_MOESM1_ESM.pdf]

Supporting information:

Continuous cardiac thermometry via simultaneous catheter tracking and undersampled radial golden angle acquisition for radiofrequency ablation monitoring

Maxime Yon[1,2,3], Marylène Delcey[1,2,3], Pierre Bour[1,2,3], William Grissom[4], Bruno Quesson[1,2,3] and Valéry Ozenne[1,2,3]

[1] IHU Liryc, Electrophysiology and Heart Modeling Institute, Foundation Bordeaux Université, F-33600 Pessac-Bordeaux, France
[2] Univ. Bordeaux, Centre de recherche Cardio-Thoracique de Bordeaux, U1045, F-33000, Bordeaux, France
[3] INSERM, Centre de recherche Cardio-Thoracique de Bordeaux, U1045, F-33000 Bordeaux, France
[4] Department of Biomedical Engineering, Vanderbilt University, 5824 Stevenson Center, Nashville, TN 37235 USA

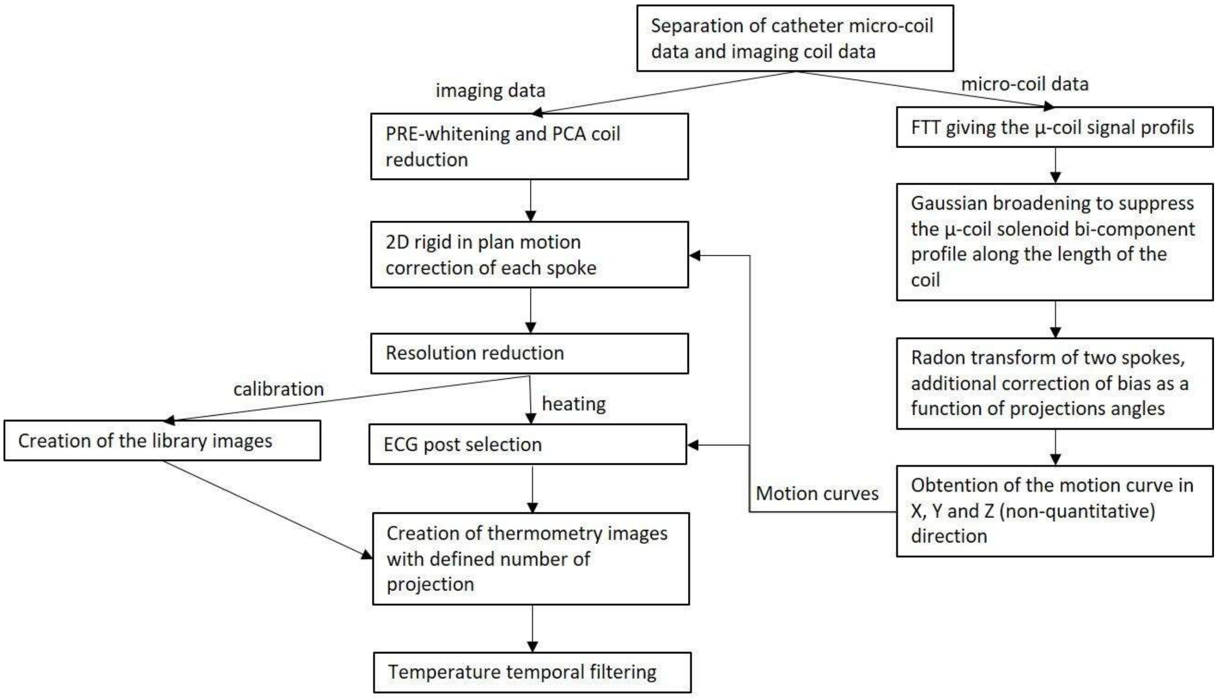

Figure 1: Flowchart of the data processing for continuous cardiac thermometry via simultaneous catheter tracking and undersampled radial golden angle acquisition

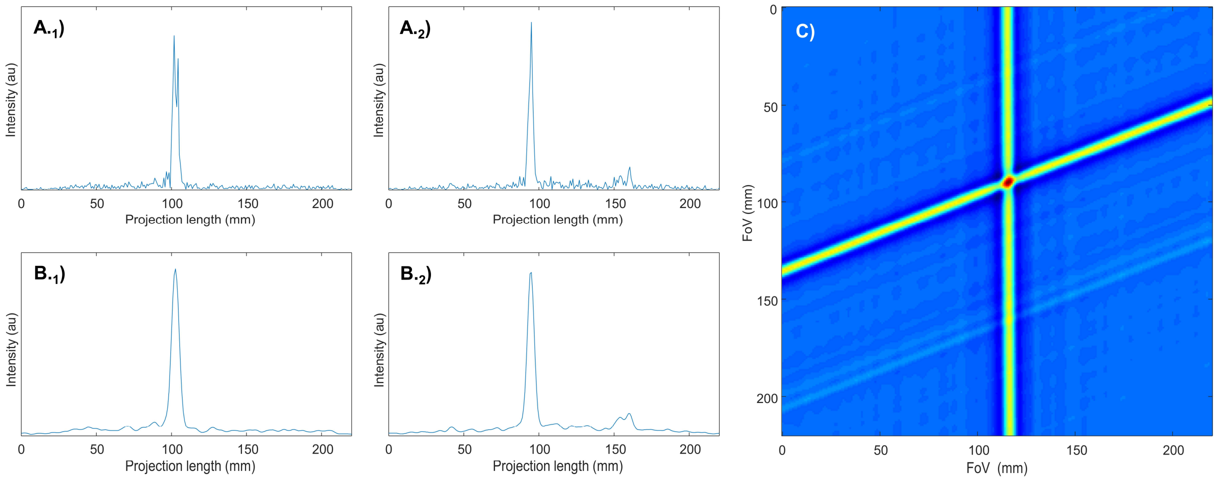

Figure 2: A.$_{1\text{-}2}$: Non-filtered consecutive projections of one catheter micro-coil signal recorded during the *in vivo* experiment on one sheep heart. The A.1 projection shows the splitting pattern characteristic of a profile recorded parallel to the solenoid length while A.2 shows a single characteristic of an orientation closer to the perpendicular of the solenoid axis. B.$_{1\text{-}2}$: Same projections after Gaussian-weighted moving average over a window of 10 points allowing smoothing the splitting pattern. C: intensity map obtained by 2D radon transform of the two projections allowing to localize the catheter micro-coil in the acquisition plan.

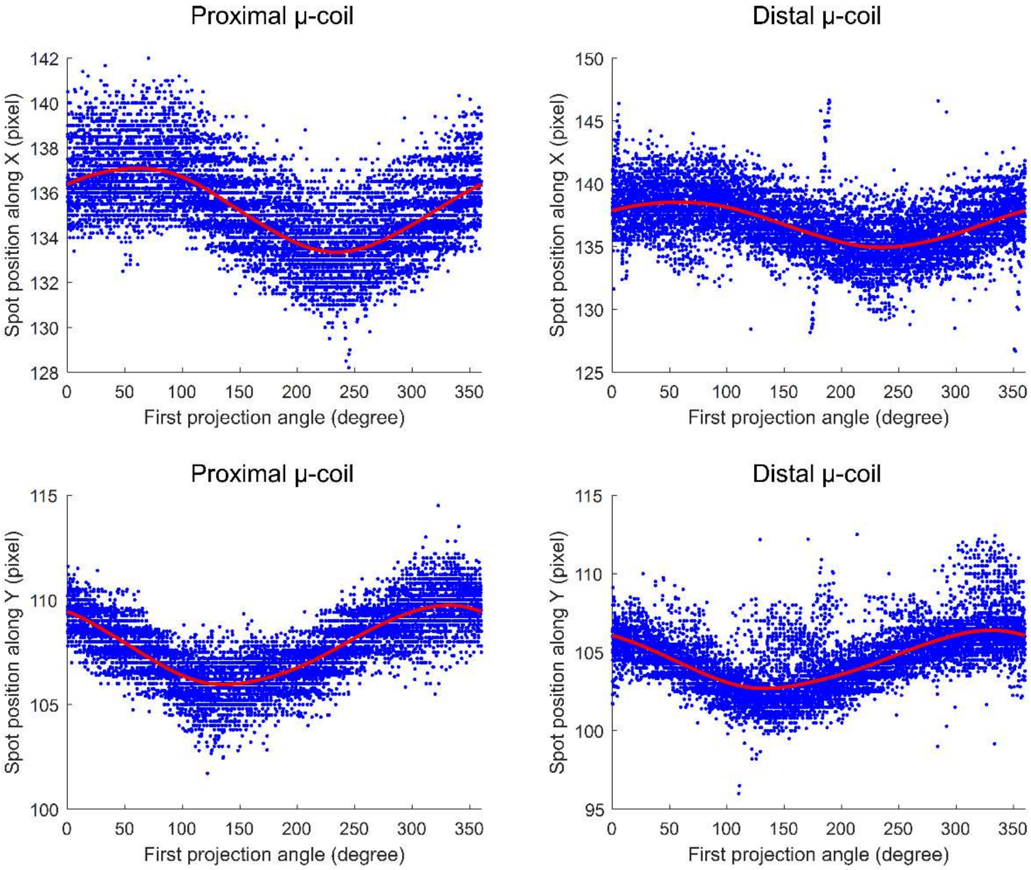

Figure 3: Plot of the two micro-coils (Proximal and distal) positions as function as the first projection angle. A bias dependent on the projection angle is clearly present and is corrected by subtraction of the fitted values presented here in red.

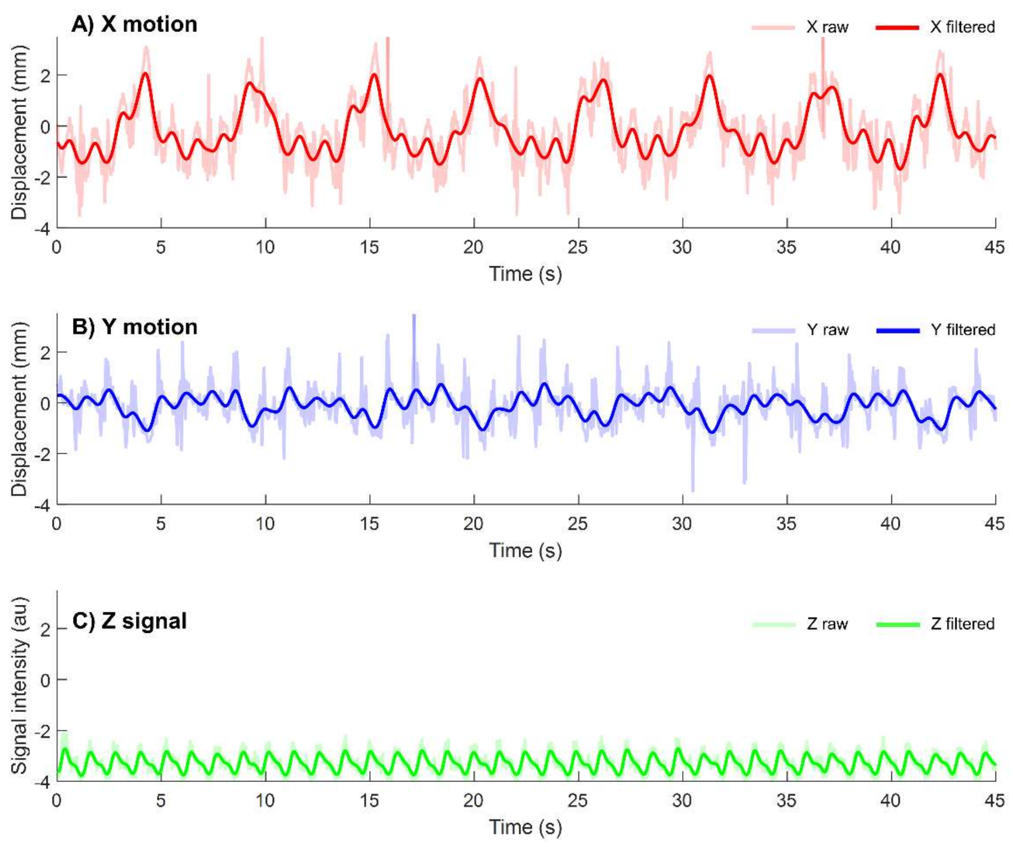

Figure 4: Overlay between the raw and filtered motions curve obtained with the catheter placed in contact with the myocardial muscle of the left ventricle in an anesthetized sheep. The temporal filtering is performed with a Gaussian filter with a cut of frequency of 0.883 Hz.

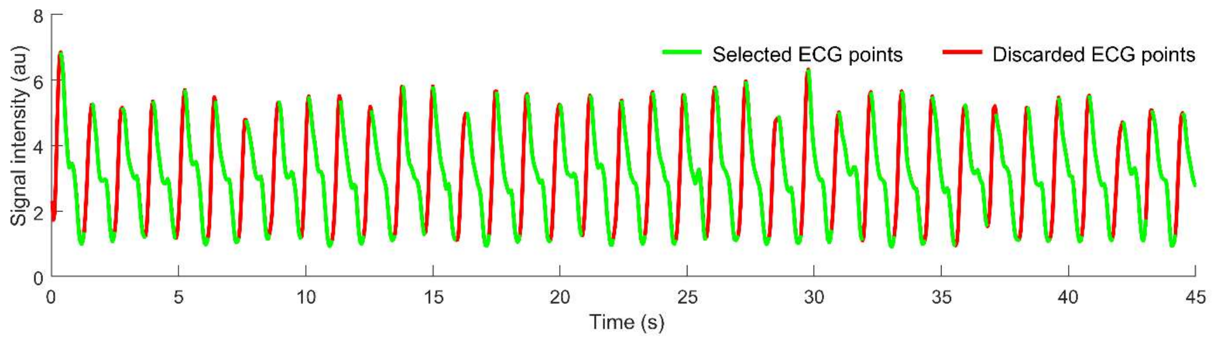

Figure 5: Plot of the micro-coil signal intensity with the selected points in green (70 %) and discarded points in red (30 %). The selection of the projection was performed to limit the first-order derivative intensity on the Z intensity and optimized based on the resulting thermometry uncertainty.

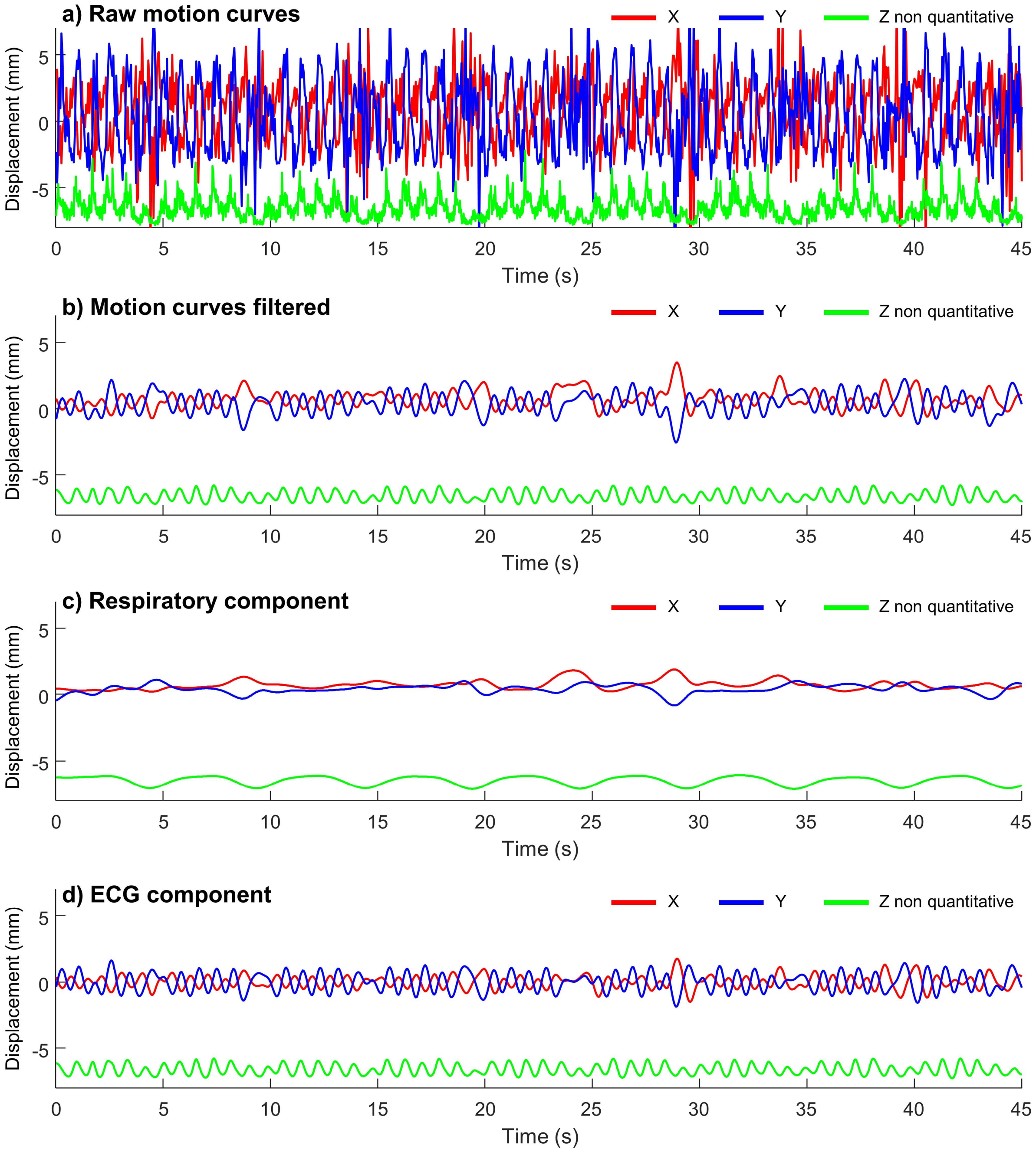

Figure 6: Motion curves obtained by following the catheter micro coils by Radon transformation of two consecutive radial projections with the catheter in contact with the myocardium of the left ventricle. The signals have a temporal resolution of two TR (≈50 ms) and the two (proximal and distal) coil signals have been averaged. a) Motion curves without any temporal filtering. b) Motion curves filtered by medium frequency Gaussian filter with a cut of frequency at 0.883 Hz. c) Respiratory component of the motion curves obtained by Gaussian low-pass temporal filtering with a cut-off frequency of 0.377 Hz (at -3dB). d) ECG component of the motion curve obtained by subtracting the respiratory component from the signal filtered by medium-frequency Gaussian. In all cases, the in-plane motion curves are mean-centered to 0 while the Z intensity curve is artificially displayed with an offset of -6.6 mm for the sake of clarity.

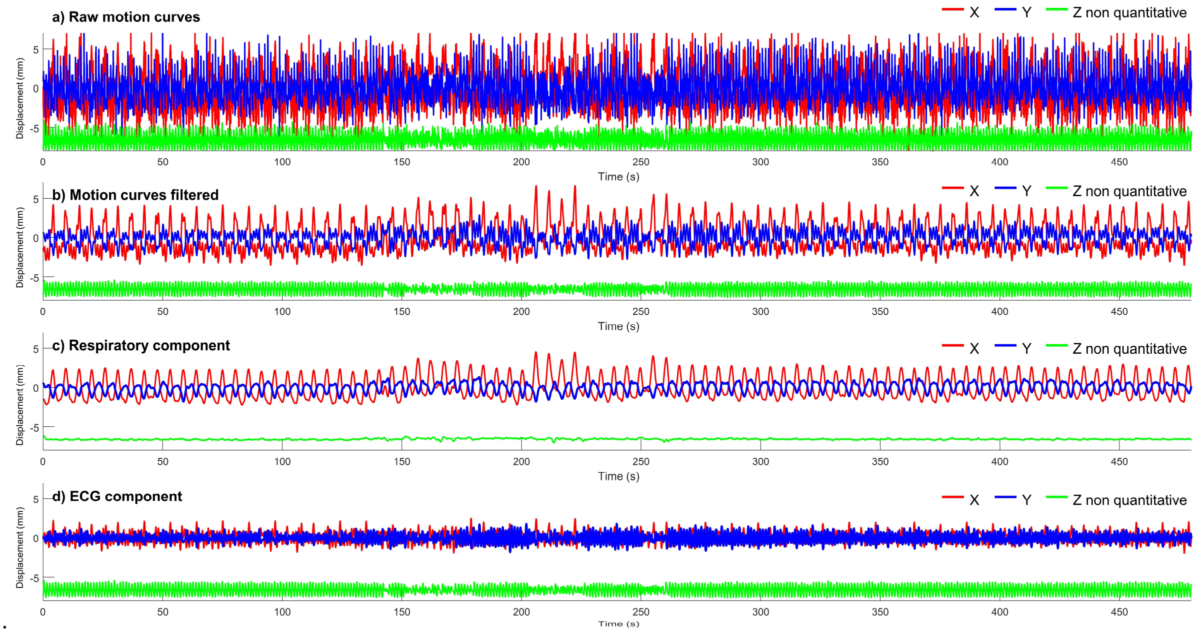

Figure 7: Entire acquisition motion curves obtained by following the catheter micro coils by Radon transformation of two consecutive radial projections with the catheter in contact with the myocardium of the left ventricle during the short axis experiment. The acquisition and processing parameters are available in the caption of figure 4. The variations of the cardiac rhythm are especially noticeable on the Z intensity signal between 140 s and 260 s.

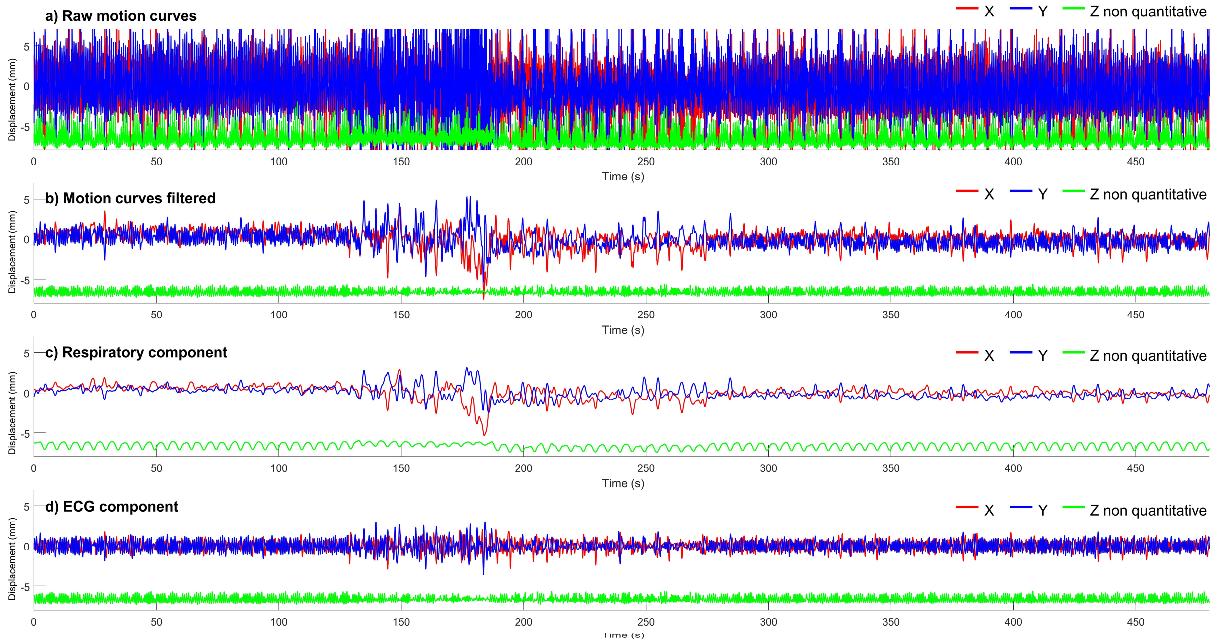

Figure 8: ***Motion curves obtained by following the catheter micro coils by Radon transformation of two consecutive radial projections with the catheter in contact with the myocardium of the left ventricle during the entire short-axis experiment.*** The acquisition and processing parameters are available in the caption of the Supporting information figure 6. The variations of the cardiac rhythm and catheter motion amplitude are especially noticeable on the X and Y motion curves signal between 130 s and 270 s.

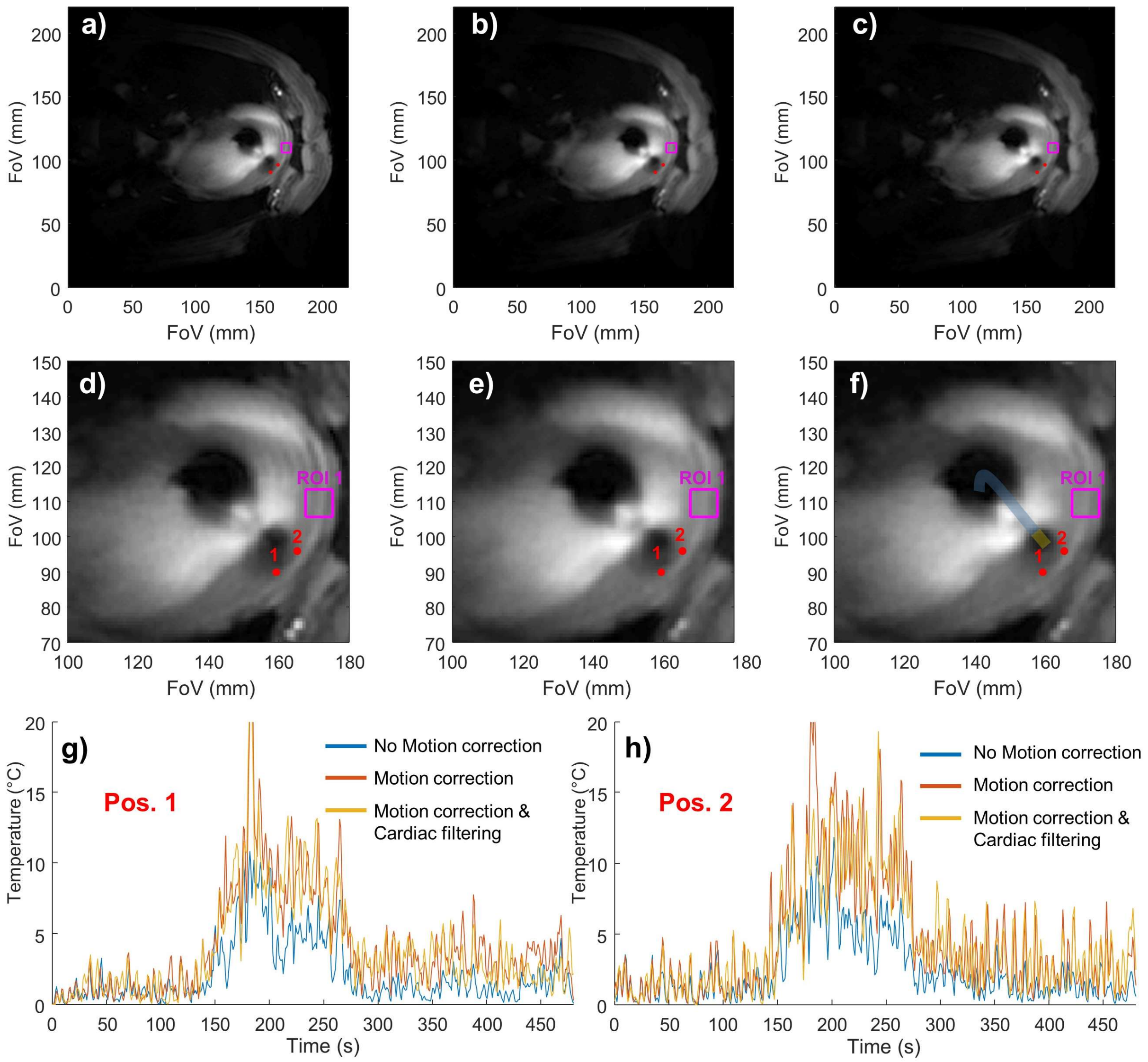

Figure 9: Radial GRE magnitude images and temperature evolution curves acquired in short-axis orientation during RF ablation. Magnitude images in full FoV (first row) and zoomed view (second row) reconstructed by NUFFT with the projections acquired during the entire eight minutes acquisition with a resolution of 0.86 × 0.86 × 3 mm^3. Panels a) and d) without motion correction; panels b) and e) with motion correction based on total filtered motion curves; panels c) and f) reconstructed with motion correction and selection of only 70 % of the projection based on cardiac cycle. A schematic representation of the approximate position of the catheter is shown in panel f. g) and h) plot temperature curves at positions 1 and 2, respectively. The mean standard deviations in the square ROI were 0.7, 1.1, and 1.2 °C for the temperature obtained without motion correction, with motion correction, and with motion correction and cardiac phase filtering, respectively.

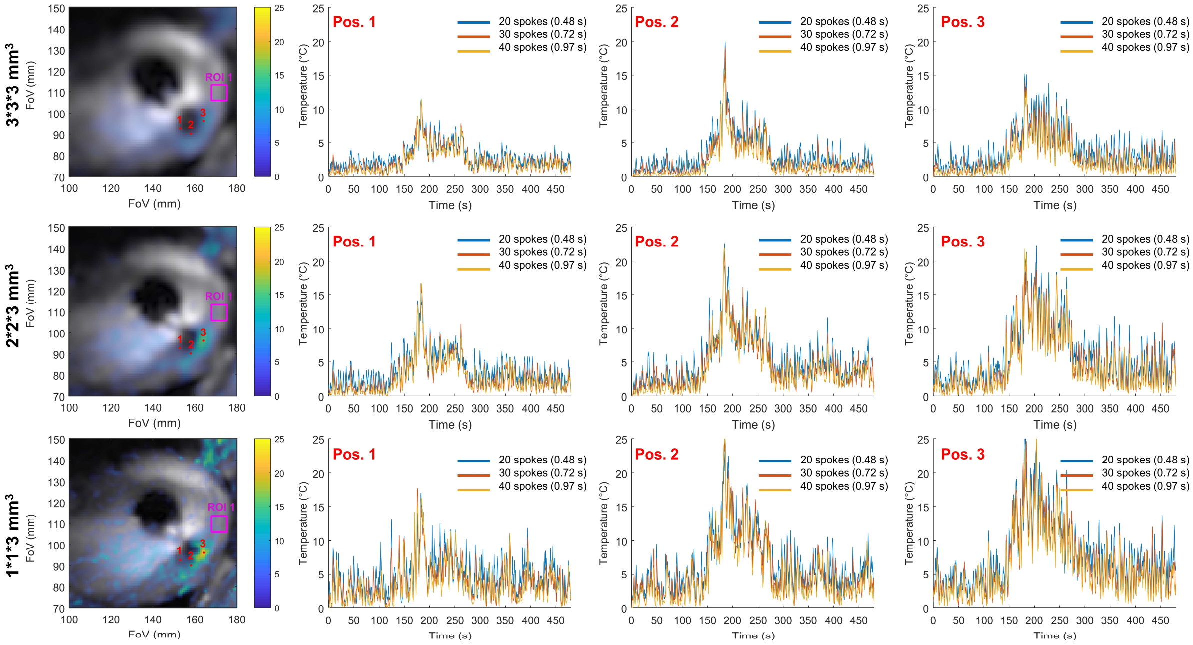

Figure 10: Influence of temporal and spatial resolution on temperature maps in the second *in vivo* experiment. left column: temperature maps obtained with 40 projections a time = 188s with in-plan resolutions of 1 × 1 mm², 2 × 2 mm² and 3 × 3mm² from top to bottom and a 3 mm slice thickness. The regularization parameters are beta = 0.05 and lambda = 0.1, 0.2 and 0.5 for the resolution 1 × 1, 2 × 2 and 3 × 3 mm², respectively. The temperature curves are presented at three spatial locations identified by the red points 1, 2, 3. The three curves: blue, red, and yellow show the results at three temporal resolutions: 0.48, 0.72, and 0.97 s corresponding respectively to the acquisition of 20, 30, and 40 consecutive projections. Each curve has been temporally filtered with a Butterworth filter with a cut of frequency of 0.14 Hz. The accelerations, the mean standard deviations in the ROIs, and the calculation times are given in table 1.

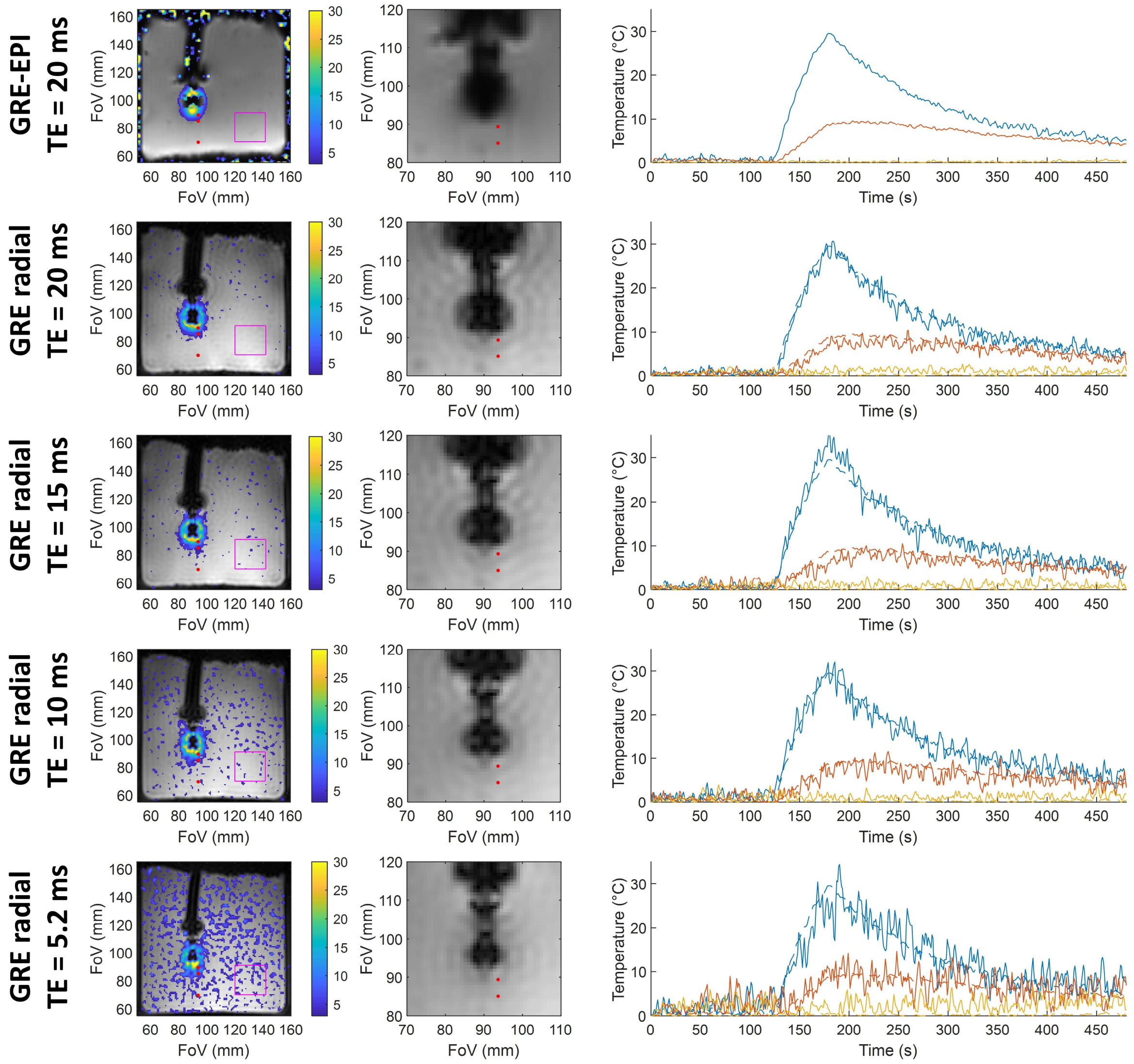

Figure 11: Comparison of magnitude images and thermometry uncertainty for GRE-EPI acquisition and radial acquisition with four different echo times: 20, 15, 10, and 5.2 ms. The temporal resolution of the thermometry maps is 1.2 seconds in EPI and 0.97 s for the four GRE radial acquisitions corresponding to 40, 50, 68, and 104 projections for TEs of 20, 15, 10, and 5 ms respectively. The GRE-EPI temperature curves are repeated in a dashed line on the GRE-radial temperature curves. The standard deviation of the temperature in the purple ROIs are: 0.19 ± 0.03 °C for the EPI acquisition and 0.53 ± 0.06 °C, 0.63 ± 0.08 °C, 0.9 ± 0.1 °C, and 1.5 ± 0.2 °C for the GRE acquisition with TE of 20, 15, 10 and 5.2 ms respectively.
